# Supplementary material for: Learning of distant state predictions by the orbitofrontal cortex in humans
Source: Nat Commun. 2019 Jun 11;10:2554. doi: 10.1038/s41467-019-10597-z (PMC6560030; doi:10.1038/s41467-019-10597-z)
Supplement: Supplementary file 1 — Supplementary Information [file 41467_2019_10597_MOESM1_ESM.pdf]

Supplementary Information

Learning of distant state predictions by the orbitofrontal cortex in humans

G. Elliott Wimmer and Christian Büchel

Supplementary Figures and Supplementary Tables

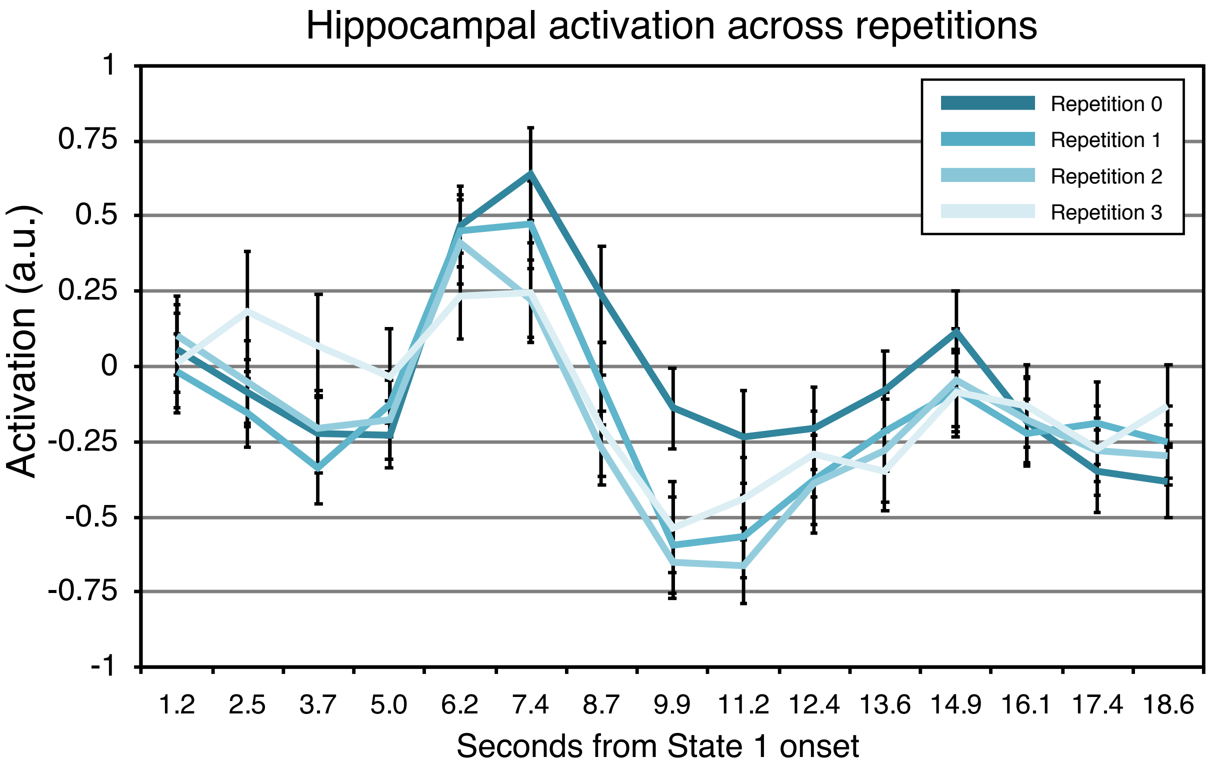

Supplementary Figure 1. Timecourse of hippocampal activity at state 1 illustrating a decrease across learning repetitions, as shown the whole-brain univariate analysis in **Figure 2**. Sequence onset is at time 0; expected peak of BOLD response to state 1 is between 6.2 – 8.7 s. (Repetition 0 in darkest aqua; repetition 3 in lightest aqua; error bars represent SEM.)

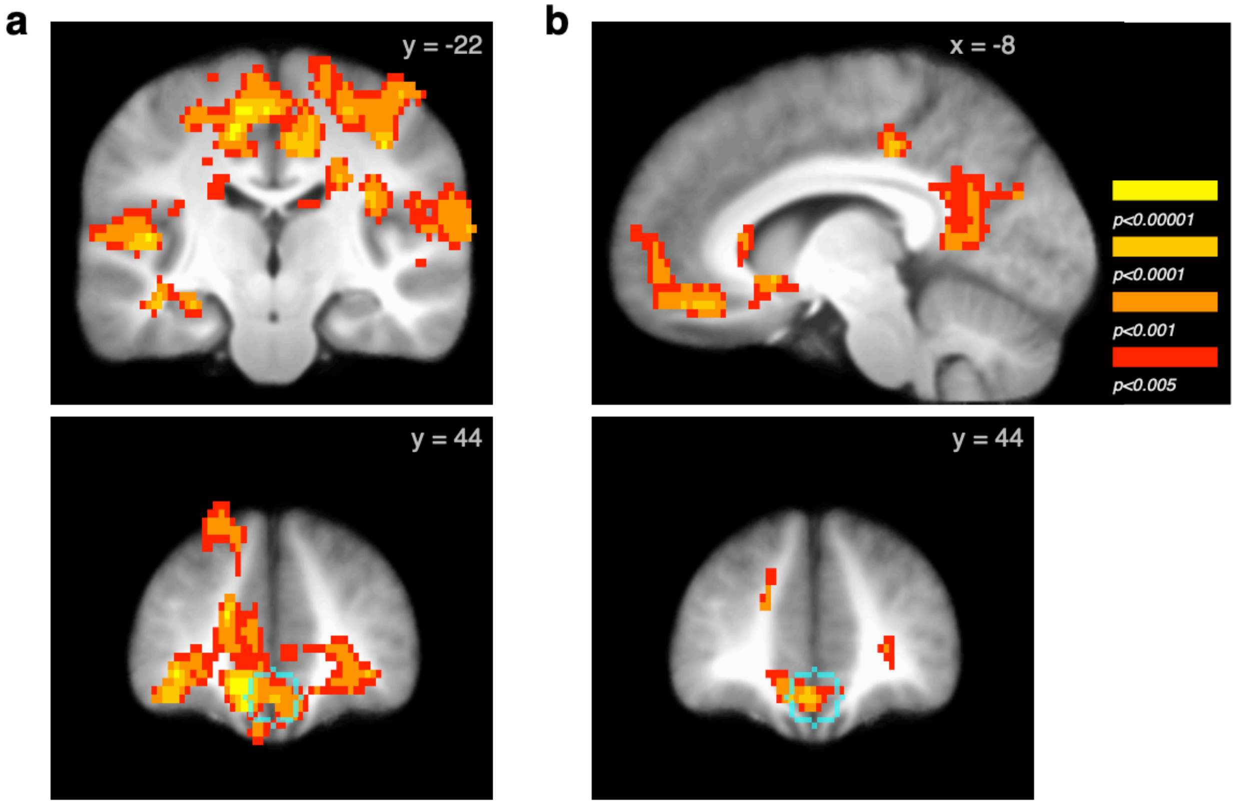

Supplementary Figure 2. Reward-related univariate responses at feedback and during post-feedback rest, related to the univariate analysis presented in **Figure 2**. (a) Reward versus loss feedback at the reward stage, including left hippocampus (top), and overlaid with the OFC-VMPFC ROI (bottom) (b) Reward versus loss feedback during the post-feedback rest period, including overlap with the OFC-VMPFC ROI in cyan (bottom). (Images $p < 0.05$ whole-brain FWE-corrected.) Full maps available at: https://neurovault.org/images/100619/ and https://neurovault.org/images/100620/

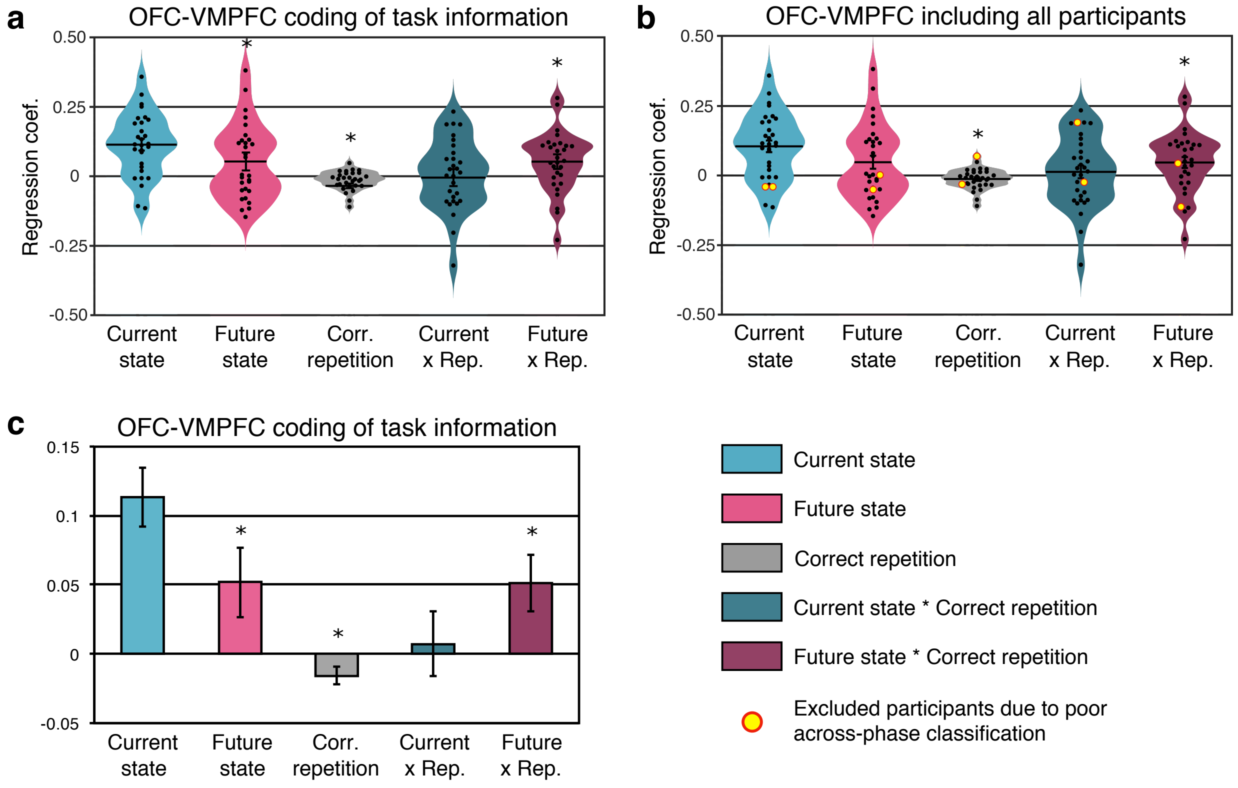

Supplementary Figure 3. OFC-VMPFC coding of task information including all participants. (a) OFC-VMPFC coding of task information as shown in **Figure 3b** for comparison. (b) OFC-VMPFC coding of task information including the 2 participants that were excluded from the primary analysis because of below-zero discrimination of actual state 1 and state 3 on-screen categories (individual datapoints marked in yellow). (c) OFC-VMPFC effects as in (a) shown in a standard column chart format instead of a violin plot. (Error bars represent SEM.)

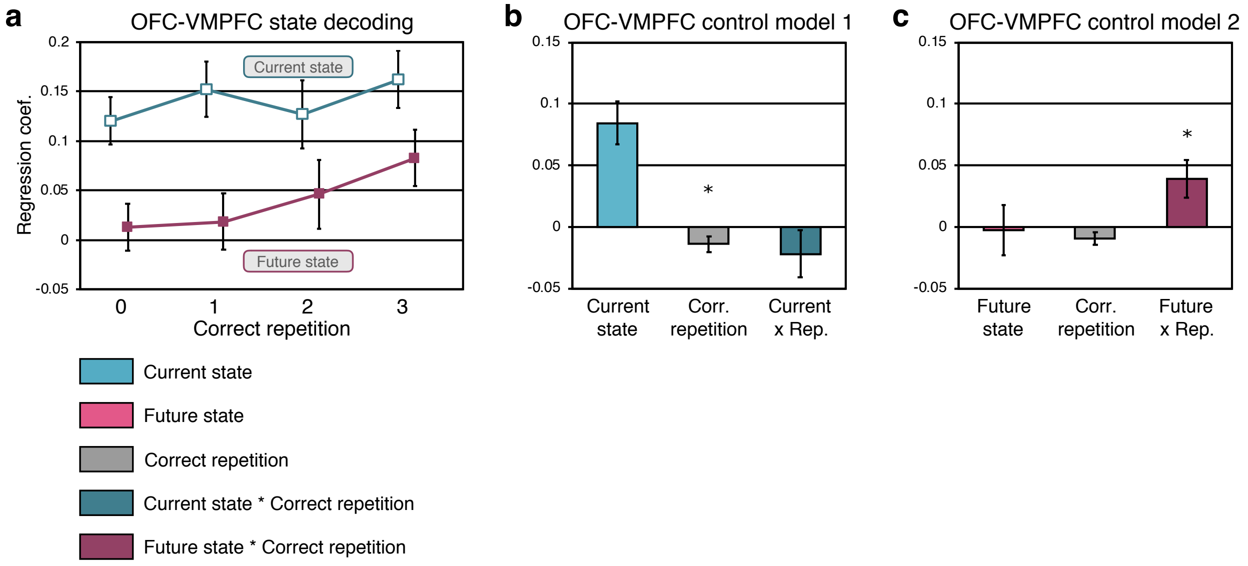

Supplementary Figure 4. State representation in the OFC-VMPFC across repetitions and control regression models. (a) Effects of current and future state modeled separately for each bin of correct repetition, as in **Figure 3c**. Current state in aqua; future state in magenta. (b) Information representing current state, correct repetition, and interaction of current state and correct repetition in a control model omitting future state. Note that effects are similar to the full model shown in Figure 3b. (c) Information representing future state, correct repetition, and the critical interaction of future state and correct repetition in a control model omitting current state. (* $p < 0.05$; ** $p < 0.01$; *** $p < 0.001$; error bars represent SEM.)

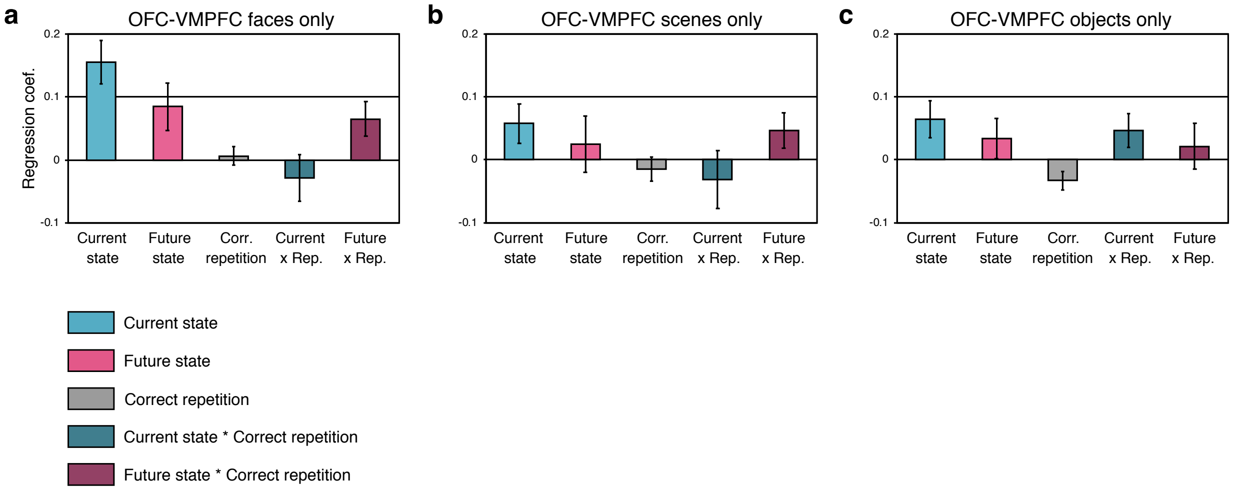

Supplementary Figure 5. Current and future state representation at state 1 in the OFC-VMPFC, as in **Figure 3b**, plotted separately for the 3 stimulus categories. (a) Information coding for face stimuli only. (b) Information coding for scene decoding only. (c) Information coding for object stimuli only. Current state in aqua; future state in magenta. (Error bars represent SEM.**)**

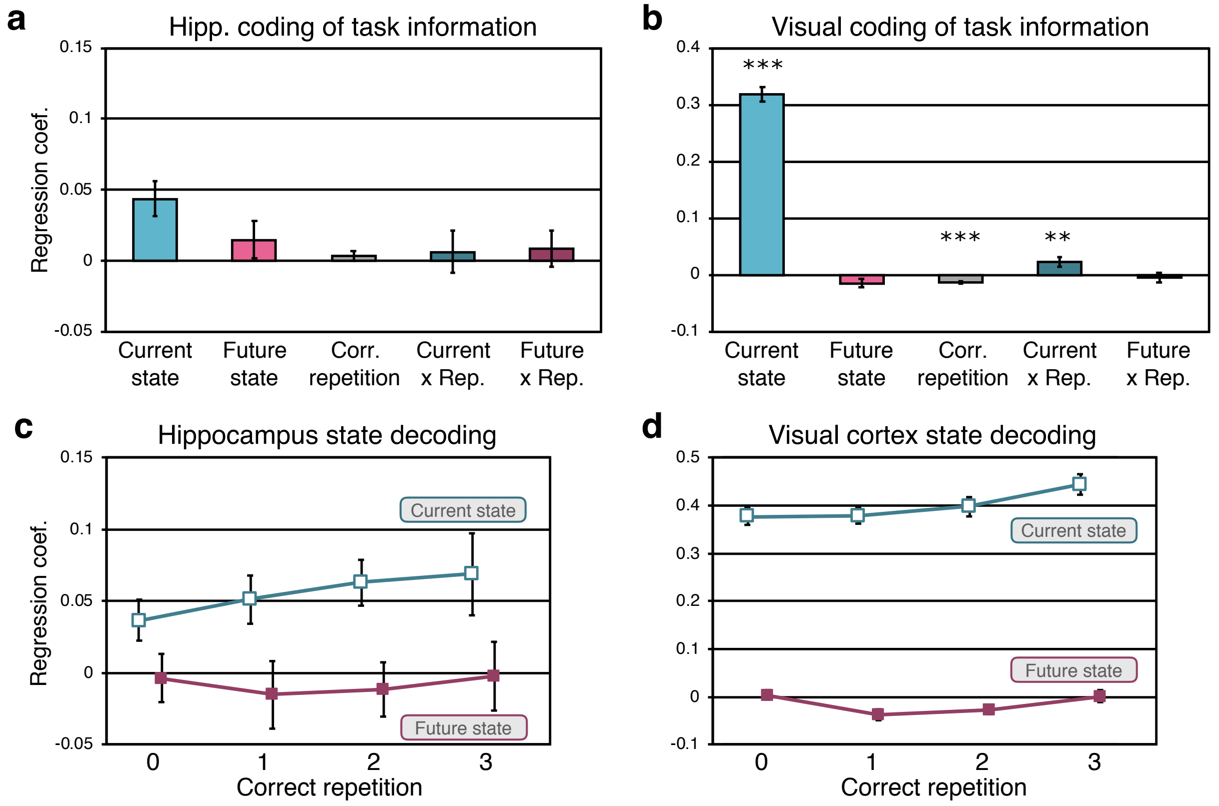

Supplementary Figure 6. Current and future state representation at state 1 in the hippocampus (a) and in visual control regions (b), as in **Figure 3b**. (c) Results from an analysis conducted separately for each correct repetition bin for illustration in the hippocampus. Current state in aqua; future state in magenta. (d) As in (c) for the visual cortex. (* $p < 0.05$; ** $p < 0.01$; *** $p < 0.001$; statistical comparisons for the hippocampus were not completed on the current state representation because the plotted data represents results after exclusion of participants with poor current state decoding. Error bars represent SEM.)

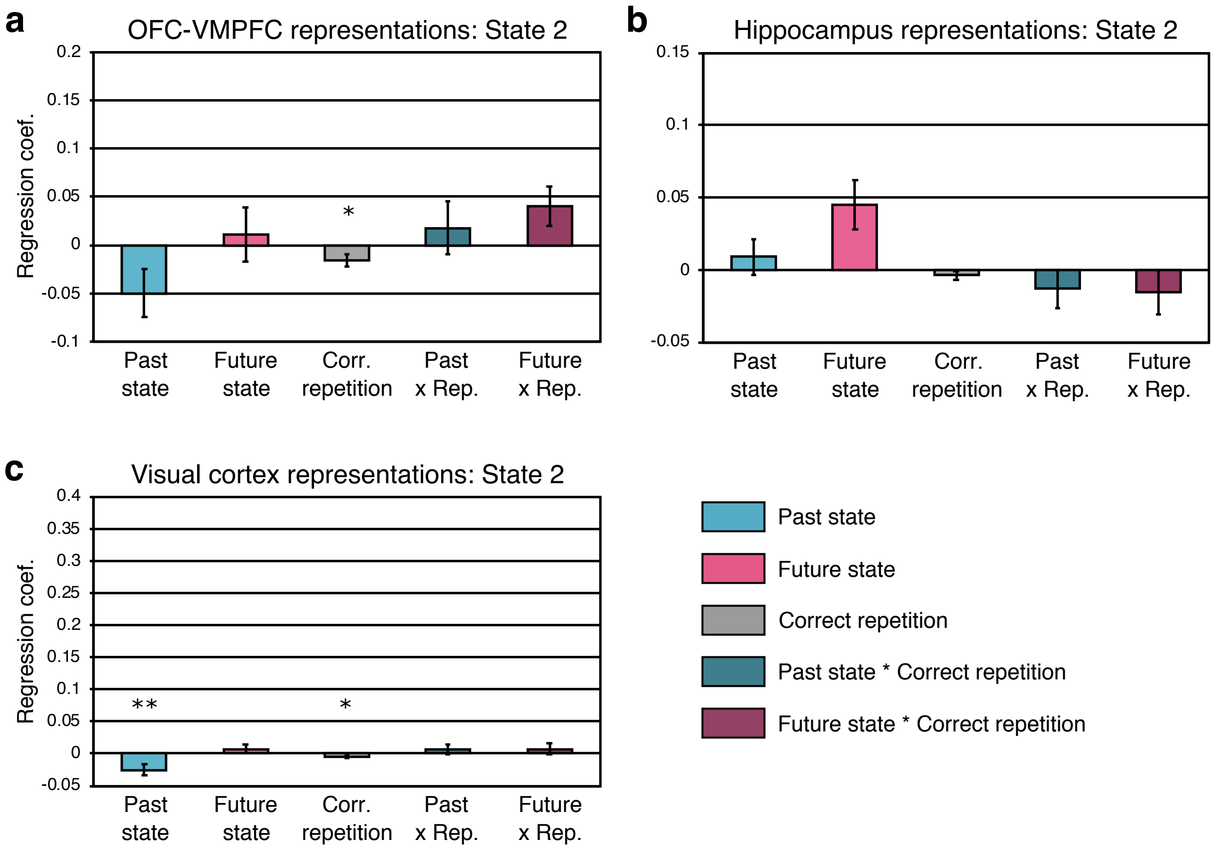

Supplementary Figure 7. State representation at state 2 in the OFC-VMPFC (a), hippocampus (b), and visual control regions (c). Note that the future by correct repetition effect in the OFC-VMPFC was positive ($p = 0.062$), similar to the effect at state 1. (* $p < 0.05$; ** $p < 0.01$; *** $p < 0.001$; error bars represent SEM.)

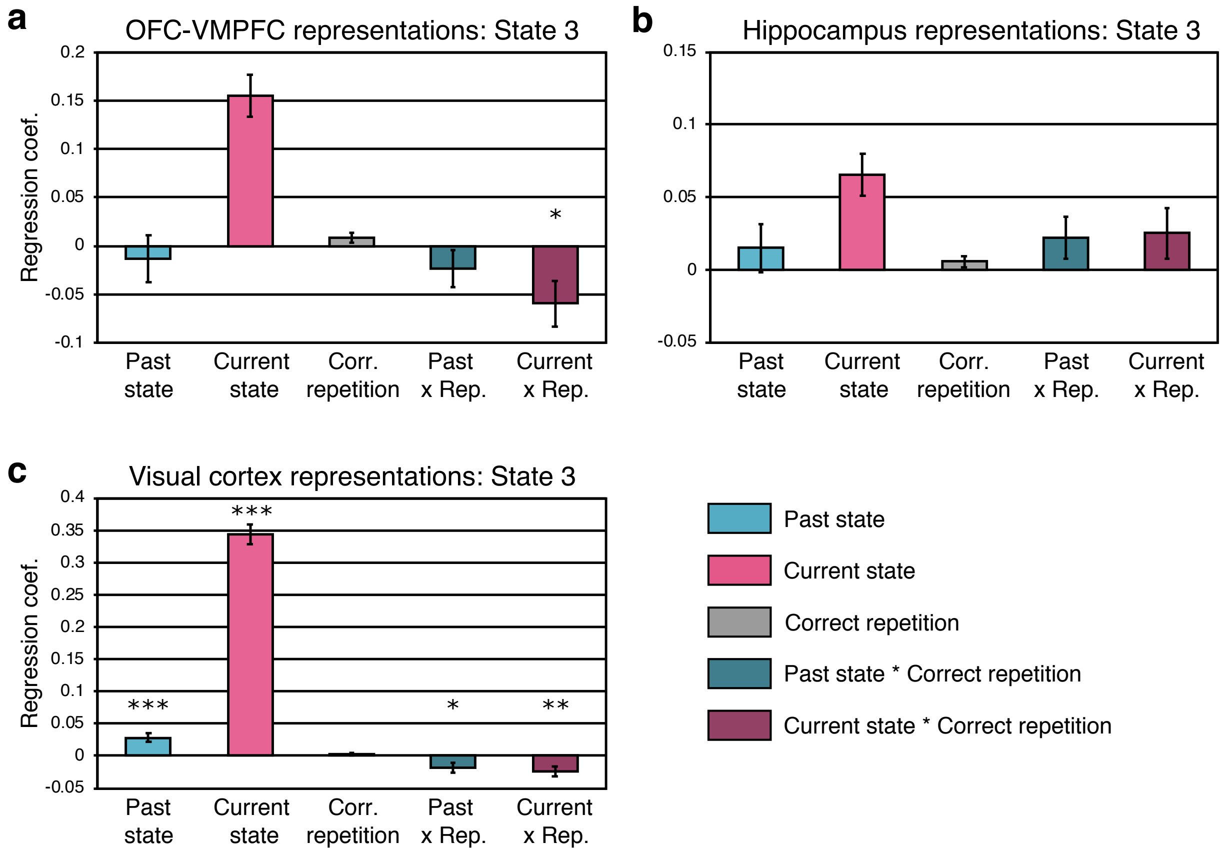

Supplementary Figure 8. State representation at state 3 in the OFC-VMPFC (a), hippocampus (b), and visual control regions (c). (* $p < 0.05$; ** $p < 0.01$; *** $p < 0.001$; statistical comparisons for OFC-VMPFC and hippocampus were not completed on the current state representation because the plotted data represents results after exclusion of participants with poor current state decoding; error bars represent SEM.)

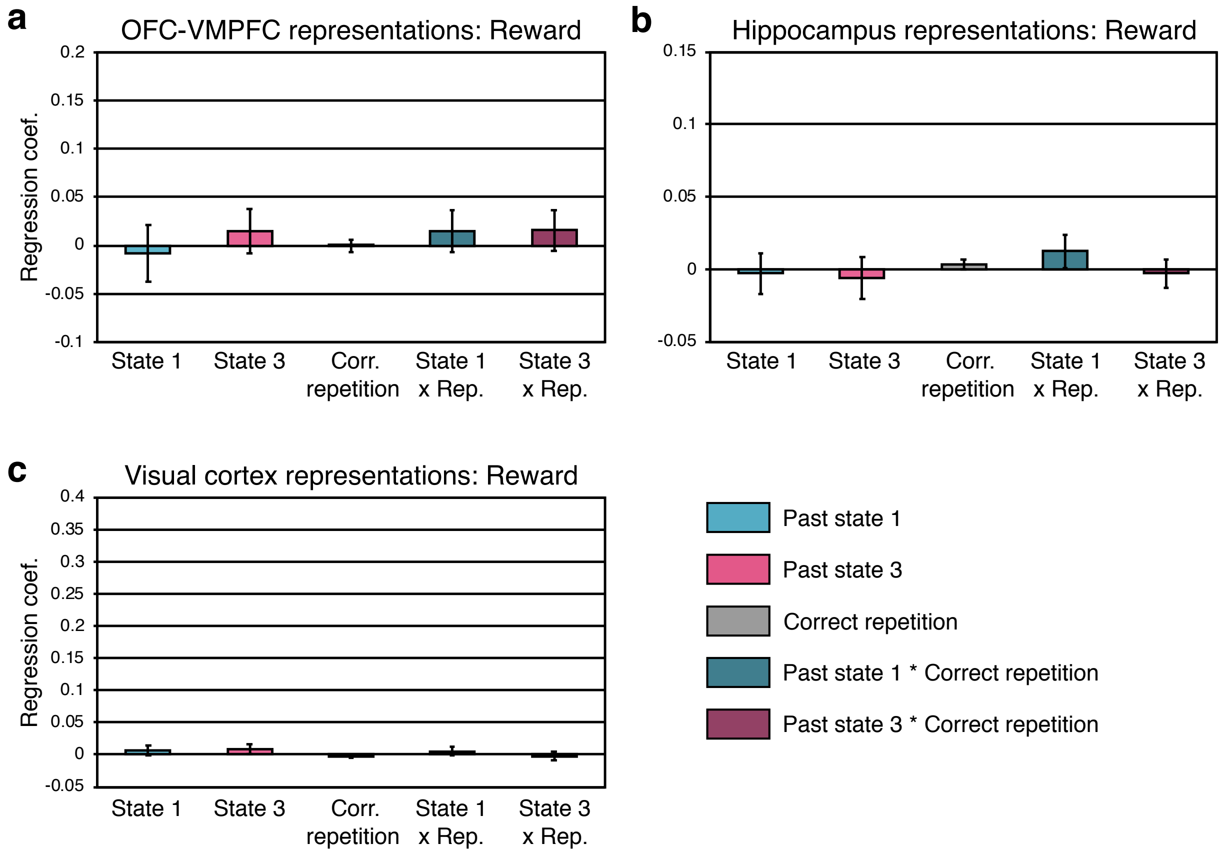

Supplementary Figure 9. State representation at the feedback state in the OFC-VMPFC (a), hippocampus (b), and visual control regions (c). (* $p < 0.05$; ** $p < 0.01$; *** $p < 0.001$; error bars represent SEM.)

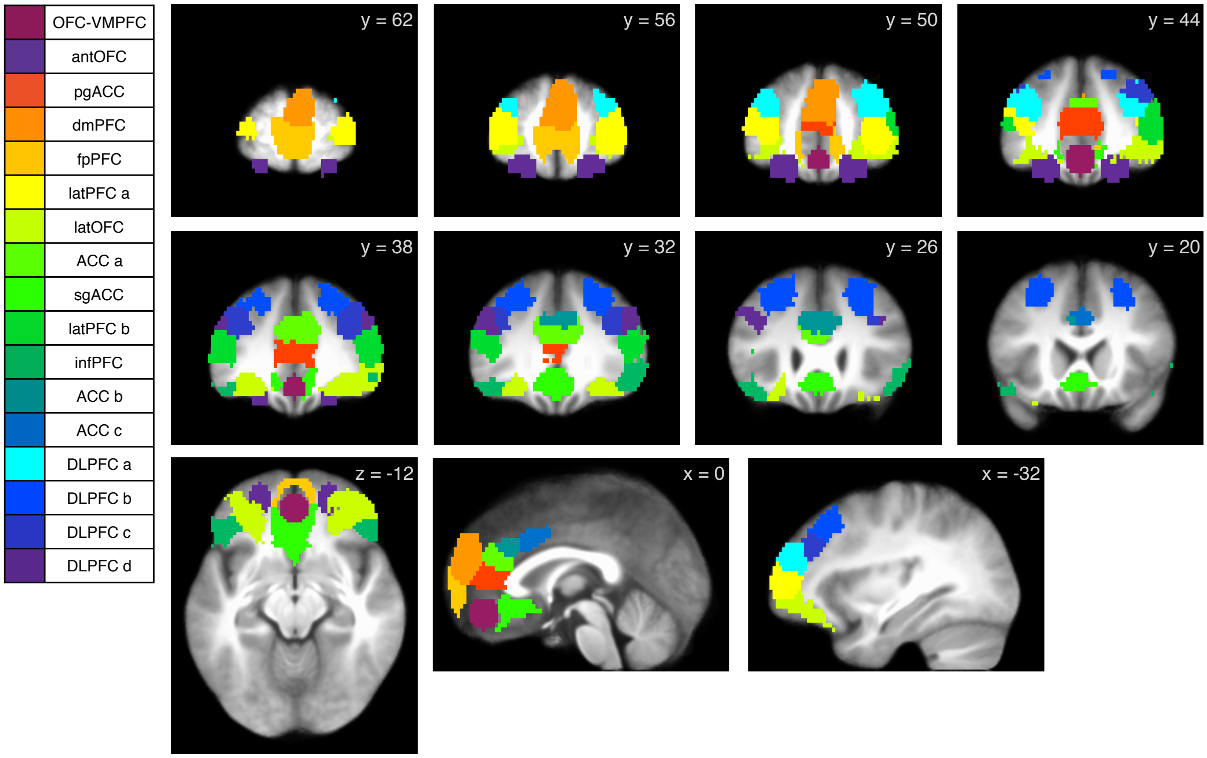

Supplementary Figure 10. Regions of interest for additional testing of task information coding in the prefrontal cortex. Regions outside of the OFC-VMPFC and anterior OFC were derived from a functional coactivation map created from Neurosynth (Chang et al., 2018). Results are shown in Table S2. Abbreviations: anterior OFC (antOFC), perigenual anterior cingulate (pgACC), dorsomedial PFC (dmPFC), frontopolar PFC (fpPFC), lateral PFC (latPFC parts a and b), lateral OFC (latOFC), ACC (ACC parts a, b, and c), subgenual ACC (sgACC), inferior PFC (infPFC), dorsolateral PFC (dlPFC parts a, b, c, and d). Full mask available at: https://neurovault.org/images/122507/

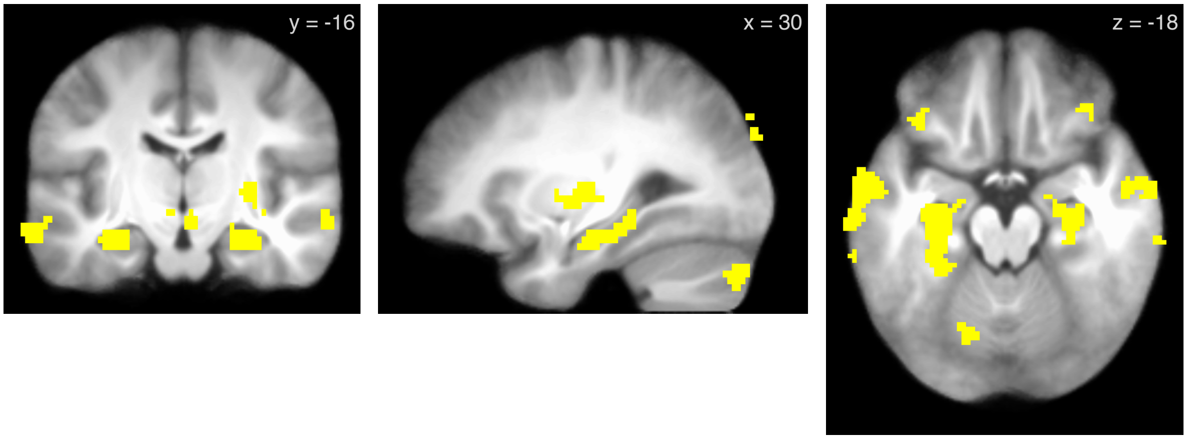

Supplementary Figure 11. Connectivity (PPI) between the OFC-VMPFC and the hippocampus: conjunction of effects at state 1, feedback state, and post-feedback rest (individual maps thresholded at $p < 0.0001$ unc.). Full maps available at: https://neurovault.org/images/123490/, https://neurovault.org/images/123491/, https://neurovault.org/images/123492/

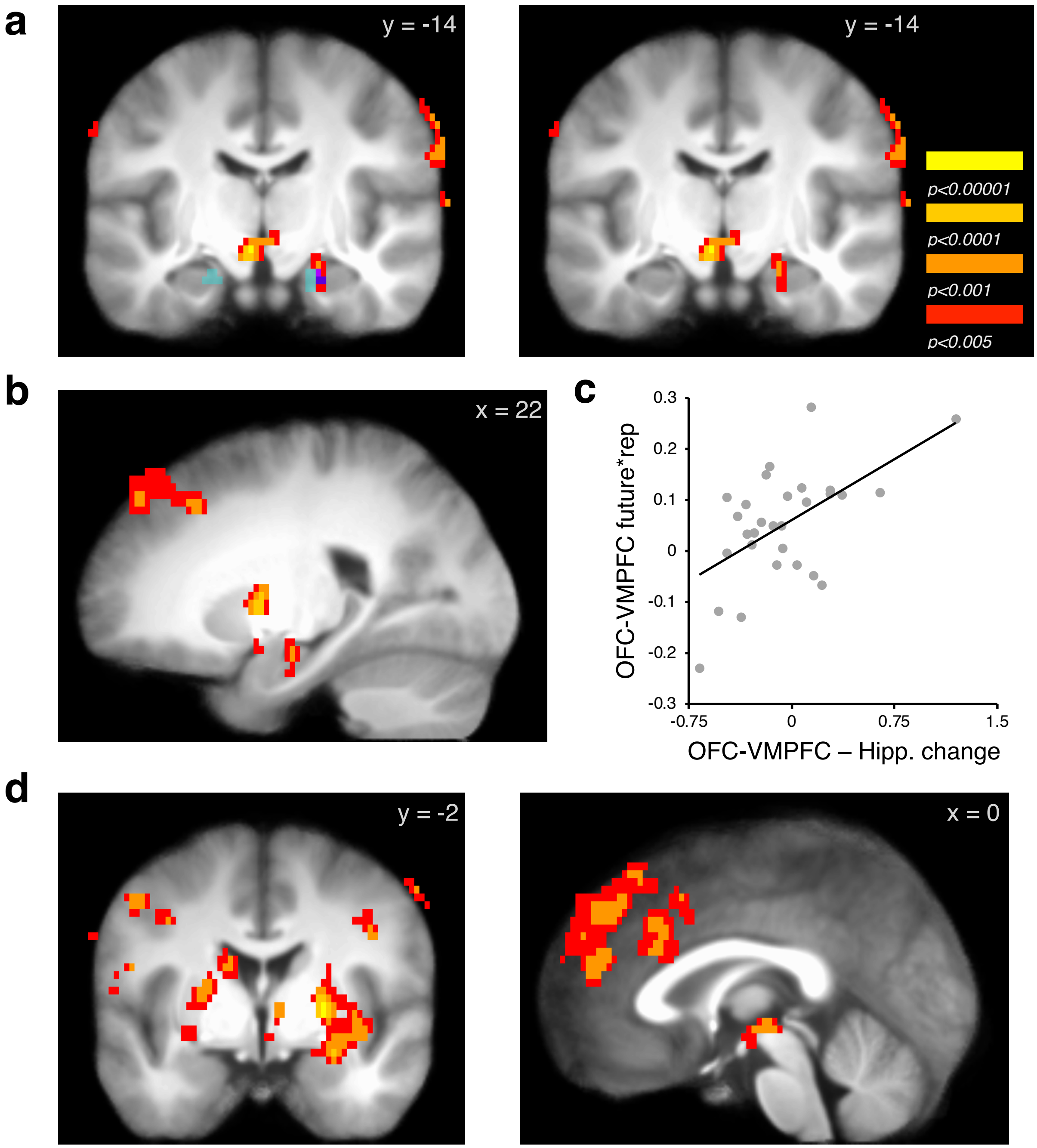

Supplementary Figure 12. Connectivity between the OFC-VMPFC and other regions for late versus early repetitions in successfully learned sequences that correlated with individual differences in the OFC-VMPFC future state by repetition effect. (a) A cluster including the hippocampus, midbrain, and thalamus ($p < 0.05$ whole-brain FWE). Blue and purple represent overlap with decay effects in hippocampus as in Figure 2 ($p<0.005$;

non-overlapping voxels in cyan). (b) Hippocampal cluster in a sagittal view. (c) Illustration of OFC-VMPFC – hippocampal correlation with OFC-VMPFC future state by repetition effect. (d) Additional clusters included the putamen as well as the dorsal medial and dorsal lateral PFC. (Images $p < 0.05$ whole-brain FWE-corrected.) Full map available at: https://neurovault.org/images/123494/

Supplementary Table 1. Summary of univariate analysis results. Clusters of activity exceeding whole-brain $p < 0.05$ FWE-corrected (cluster-forming threshold $p < 0.005$). Within each cluster, the first 10 regions are listed that include >= 10 voxels in a cluster. For the reward versus non-reward contrast at feedback, the cluster-forming threshold was set to $p < 0.0005$ to allow for informative clusters.

Contrast	Regions	Cluster size	x	y	z	Peak z stat
Decay (State 1)	L Superior Frontal Gyrus	454	-30	26	54	3.78
	R Middle Occipital Gyrus<br>R Middle Temporal Gyrus<br>R Superior Occipital Gyrus	450	18	-100	18	4.1
	R Lingual Gyrus<br>R Fusiform Gyrus<br>R Middle Occipital Gyrus	295	24	-78	-9	3.96
	R Parahippocampal Gyrus<br>R Fusiform Gyrus<br>R Occipital Gyrus	218	28	-44	-12	4.24
	L Middle Occipital Gyrus<br>L Superior Occipital Gyrus	126	-34	-86	18	3.42
Decay (State 2)	R Precuneus<br>L Precuneus	296	8	-66	48	4.11
Decay (State 3)						
Reward	Middle Occipital Gyrus<br>Middle Temporal Gyrus<br>Lingual Gyrus<br>Cuneus<br>L Superior Temporal Gyrus<br>Inferior Temporal Gyrus<br>Inferior Parietal Lobule<br>Inferior Occipital Gyrus<br>R Precuneus<br>R Fusiform Gyrus<br>L Insula	5642	7.01	46	-62	6
	Medial Frontal Gyrus<br>Anterior Cingulate<br>L Middle Frontal Gyrus<br>Superior Frontal Gyrus<br>L Inferior Frontal Gyrus<br>Ventral Striatum	1855	5.24	-14	56	9

	Caudate Head<br>Rectal Gyrus<br>Subcallosal Gyrus					
	Cingulate Gyrus<br>Paracentral Lobule<br>Medial Frontal Gyrus<br>Precentral Gyrus<br>Precuneus<br>Postcentral Gyrus	1785	5.04	-10	12	27
	R Superior Temporal Gyrus<br>R Inferior Parietal Lobule<br>R Precentral Gyrus<br>R Postcentral Gyrus<br>R Insula<br>R Transverse Temporal Gyrus<br>R Inferior Frontal Gyrus	1408	5.55	58	-36	15
	L Precentral Gyrus<br>L Transverse Temporal Gyrus<br>L Postcentral Gyrus<br>L Superior Temporal Gyrus<br>L Insula	239	4.55	-44	-24	9
	R Dorsal Caudate<br>White Matter	176	4.87	22	10	30
	R Precentral Gyrus<br>R Postcentral Gyrus	168	4.35	40	-22	42
	R Putamen<br>R Insula	132	4.39	32	0	3
	R Medial Frontal Gyrus<br>R Middle Frontal Gyrus<br>R Superior Frontal Gyrus	118	4.45	16	54	3
	L Caudate Tail<br>L Posterior Putamen	86	4.35	-18	-2	27
	L Superior Temporal Gyrus	82	4.71	-64	-10	3
	L Cingulate Gyrus<br>L Precuneus	51	4.41	-8	-42	30
Reward (State rest)	L Middle Temporal Gyrus<br>Posterior Cingulate<br>Cuneus<br>Precuneus<br>L Middle Occipital Gyrus<br>Cingulate Gyrus<br>L Superior Occipital Gyrus<br>L Lingual Gyrus	1953	4.53	-20	-74	27

L Inferior Occipital Gyrus<br>L Parahippocampal Gyrus<br>L Superior Temporal Gyrus					
Medial Frontal Gyrus<br>Anterior Cingulate<br>Ventral Striatum<br>Caudate Head<br>Rectal Gyrus<br>L Superior Frontal Gyrus	1321	5.35	8	24	3
R Middle Occipital Gyrus<br>R Inferior Occipital Gyrus<br>R Cuneus<br>R Middle Temporal Gyrus<br>R Inferior Temporal Gyrus<br>R Posterior Cingulate<br>R Lingual Gyrus	995	4.53	32	-62	9
L Cingulate Gyrus<br>White Matter	458	4.76	-22	-26	36
R Precentral Gyrus<br>R Postcentral Gyrus	333	4.25	30	-38	72
R Hippocampus<br>R Parahippocampal Gyrus	250	4.93	32	-34	0
L Parahippocampal Gyrus<br>L Fusiform Gyrus<br>L Hippocampus<br>L Middle Occipital Gyrus<br>L Middle Temporal Gyrus	250	3.88	-38	-52	-12
R Precentral Gyrus<br>R Insula<br>R Postcentral Gyrus	207	4.3	56	0	18
L Medial Frontal Gyrus<br>L Superior Frontal Gyrus	181	4	-16	42	24

Supplementary Table 2. Additional PFC region of interest state information analysis. Regression results are shown for current state (state 1) and the future state (state 3) by correct repetition interaction. Results from the OFC-VMPFC are as shown in **Figure 3**. Number of included participants per ROI for the future by repetition analysis depends on exclusions for below-zero classification of state 1 and state 3. antHipp = anterior hippocampus; postHipp = posterior hippocampus.

region	current *(n = 31)*	CI	*t*-stat	*p*-value	n	fut*rep	CI	*t*-stat	*p*-value
**OFC**	**0.092**	**0.05 0.13**	**4.48**	**0.0001**	**29**	**0.051**	**0.01 0.09**	**2.53**	**0.0172**
R antOFC	-0.001	-0.04 0.03	-0.08	0.9384	22	0.011	-0.05 0.07	0.37	0.7176
L antOFC	0.014	-0.03 0.05	0.70	0.4880	24	0.033	-0.01 0.08	1.46	0.1570
pgACC	0.022	-0.01 0.06	1.27	0.2152	24	0.034	-0.01 0.08	1.57	0.1311
dmPFC	0.138	0.1 0.17	7.81	0.0000	30	0.017	-0.02 0.05	0.90	0.3749
fpPFC	0.097	0.06 0.13	5.63	0.0000	30	0.001	-0.04 0.04	0.08	0.9371
R latPFC a	0.038	0 0.08	1.95	0.0610	25	0.002	-0.05 0.05	0.08	0.9351
L latPFC a	0.021	-0.01 0.06	1.22	0.2324	21	-0.007	-0.05 0.04	-0.31	0.7588
R latOFC	0.084	0.06 0.11	7.84	0.0000	29	-0.004	-0.03 0.02	-0.30	0.7701
L latOFC	0.038	0.01 0.07	2.57	0.0153	27	-0.008	-0.04 0.03	-0.47	0.6439
ACC a	-0.007	-0.04 0.03	-0.42	0.6808	23	0.014	-0.05 0.07	0.47	0.6401
sgACC	0.017	-0.01 0.05	1.13	0.2688	27	0.008	-0.03 0.04	0.43	0.6739
R latPFC b	0.121	0.08 0.16	5.78	0.0000	28	-0.007	-0.04 0.03	-0.38	0.7100
L latPFC b	0.093	0.04 0.14	3.90	0.0005	29	-0.026	-0.09 0.04	-0.86	0.3952
R infPFC	0.052	0 0.1	2.16	0.0393	29	-0.003	-0.06 0.06	-0.11	0.9134
L infPFC	0.061	0.01 0.11	2.57	0.0153	28	0.052	-0.02 0.12	1.45	0.1573
ACC b	0.020	-0.02 0.06	0.98	0.3357	29	-0.018	-0.07 0.03	-0.76	0.4509
ACC c	0.020	-0.04 0.08	0.70	0.4889	25	0.008	-0.05 0.07	0.27	0.7931
R DLPFC a	0.050	0 0.1	2.04	0.0507	27	0.050	-0.01 0.11	1.79	0.0858
L DLPFC a	0.025	-0.01 0.06	1.50	0.1451	25	0.027	-0.06 0.11	0.67	0.5115
R DLPFC b	0.027	0 0.06	1.88	0.0694	24	0.035	-0.01 0.08	1.58	0.1278
L DLPFC b	0.051	0.02 0.08	3.33	0.0023	**24**	**0.047**	**0.02 0.08**	**3.10**	**0.0051**
R DLPFC c	-0.021	-0.07 0.02	-1.00	0.3232	26	0.025	-0.04 0.09	0.79	0.4377
L DLPFC c	0.076	0.02 0.13	2.66	0.0125	21	0.041	-0.03 0.11	1.28	0.2164
R DLPFC d	0.056	-0.02 0.13	1.59	0.1220	25	-0.030	-0.13 0.07	-0.64	0.5267
L DLPFC d	0.062	0.01 0.11	2.62	0.0136	27	0.070	-0.01 0.15	1.87	0.0725

	R antHipp	0.031	0.00 0.06	2.40	0.0228	30	0.001	-0.03 0.03	0.08	0.9370
	L antHipp	0.012	-0.04 0.07	0.45	0.6553	26	-0.001	-0.06 0.05	-0.06	0.9564
	R postHipp	0.038	-0.00 0.08	1.87	0.0717	27	-0.026	-0.09 0.04	-0.84	0.4091
	L postHipp	-0.013	-0.06 0.04	-0.55	0.5842	18	-0.012	-0.07 0.05	-0.43	0.6745

Supplementary Table 3. Summary of OFC-VMPFC Late > Early repetition PPI analysis effects correlated with the OFC-VMPFC future state by correct repetition effect. State 1 was the timepoint of interest; later states were analyzed in control models. The PPI contrast of late versus early repetitions (3 and 4 versus 1 and 2) or the reverse was regressed against individual differences in the OFC-VMPFC future state by correct repetition regression coefficient (as depicted in **Figure 3b**). No main effects of the contrasts of late > early repetitions or early > late repetitions were found. Clusters of activity exceed whole-brain $p < 0.05$ FWE-corrected (cluster-forming threshold $p < 0.005$). Within each cluster, the first 10 regions are listed that include >= 10 voxels in a cluster.

Contrast	Regions	Cluster size	x	y	z	Peak z stat	P-value
State 1<br>OFC PPI<br>Late > Early<br>future*repcorr	Bilat. Middle Frontal Gyrus<br>Bilat. Inferior Frontal Gyrus<br>Bilat. Superior Frontal Gyrus<br>Medial Frontal Gyrus<br>Cingulate Gyrus<br>Precentral Gyrus<br>L Putamen<br>Anterior Insula<br>L Caudate Body	5712	42	12	39	4.93	0
	R Putamen<br>R Pallidum<br>R Insula<br>R Amygdala	275	24	-2	0	4.54	0.006
	Midbrain<br>Thalamus<br>R Hippocampus	285	-6	-10	-9	4.47	0.005
	L Postcentral Gyrus<br>L Supramarginal Gyrus<br>L Inferior Parietal Lobule	282	-50	-20	24	3.99	0.005
State 1<br>OFC PPI<br>Early > Late<br>future*repcorr	–						

State 2<br>OFC PPI<br>Late > Early<br>future*repcorr	–
State 2<br>OFC PPI<br>Early > Late<br>future*repcorr	–
State 3<br>OFC PPI<br>Late > Early<br>future*repcorr	–
State 3<br>OFC PPI<br>Early > Late<br>future*repcorr	–
State reward<br>OFC PPI<br>Late > Early<br>future*repcorr	–
State reward<br>OFC PPI<br>Early > Late<br>future*repcorr	–
